# Bacterial distribution in Twilight zone of the Indian sector of Southern Ocean: V3- V4 rDNA hypervariable region data

**DOI:** 10.1016/j.dib.2022.108834

**Published:** 2022-12-16

**Authors:** Alok K. Sinha, Bhaskar V. Parli, N. Anilkumar

**Affiliations:** a Ocean Science Group, National Centre for Polar and Ocean Research, Ministry of Earth Sciences, Headland Sada, Goa 403804, India; b O’ Smart, Hydrothermal Vent Group, National Centre for Polar and Ocean Research, Ministry of Earth Sciences, Headland Sada, Goa 403804, India

**Keywords:** Metagenomics, Bacterial diversity, Southern ocean, Deep water

## Abstract

Twilight zones in oceans represent the oceanic waters between 200 m to 1000 m in depth, wherein sunlight is diffused and intensity is <1% of surface value. The activities and diversity of marine micro-organisms in this unique zone are understudied, especially in the Indian Sector of the Southern Ocean. For a better understanding of the microbial environment and diversity in the twilight zone of the Indian sector of Southern Ocean, samples were collected from 200m depth in eddy-influenced waters of Subtropical Front (STF), Sub-Antarctic Front (SAF), Polar Front (PF), waters off Kerguelen (Kw), and Prydz Bay (Pb) waters. In this article, next-generation sequencing (NGS) based amplicon data of 16s rDNA bacterial samples are presented. Hypervariable V3-V4 regions were sequenced using Hiseq platform, and data was processed using Mothur v 1.48.0, and database Silva 138.1nr. Total of nine different phyla is reported from the Southern Ocean at 200m, whereas at order level Synechococcales was found in STF waters only and SAR 11_ Clades were present in all stations.

**Specifications Table**

**Table:** 

Subject	Environmental Science
Specific subject area	Microbiology
Type of data	Table Graph Figure
How the data were acquired	Samples were collected using Niskin bottle (10 L sampler; Seabird Inc., USA) attached to CTD rosette equipped with Sea bird CTD system (SBE911 plus, Sea-Bird Electronics, USA). Five litres (5 L) of water samples were filtered through 0.22-µm pore size, 47 mm diameter polycarbonate filters (Merck Millipore, USA). DNA was isolated using Power Water DNA kit (MoBio; USA) and sequencing was carried out using primer *341 F: 5’* CCTACGGGAGGCAGCAG *3’* and *806 R: 5′* GGACTACHVGGGTTCTAAT *3’* with Hiseq Rapid V2 Kit.
Data format	Raw (All data were submitted in Fastq (Zip file) format)
Description of data collection	Amplicon data, hypervariable region (V3-V4) of bacterial 16S rDNA from the twilight zone of Southern Ocean.
Data source location	**Institution**: National Centre for Polar and Ocean Research **City/Town/Region**: Vasco da Gama **Country**: India Sea water samples were collected from 200 m depth at seven different stations from the Indian Sector of the Southern Ocean during an expedition onboard *SA Agulhas* (details in Table 1).
Data accessibility	The sequence has been submitted to the public repository Repository name: National Center for Biotechnology Information Data identification number: PRJNA878484 https://www.ncbi.nlm.nih.gov/bioproject/PRJNA878484
Related research article	Alok K. Sinha, Bhaskar V. Parli, N. Anilkumar Bacterial distribution in the Equatorial Indian Ocean using Amplicon sequencing of V3-V4 rDNA hypervariable region data. Data in Brief 45 (2022) 108673. https://doi.org/10.1016/j.dib.2022.108673 2352-3409

## Value of the Data

• First set of metagenomic data from the twilight waters of ISO. This set of data from an under-reported oceanic regime shall add to the global databases like BacDive, Planet Microbe, etc..
• Twilight zone is a hotspot for microbial activity and diversity; reported dataset can be used in linking microbial processes (enzymatic and biogeochemical processes) to diversity.
• ISO serves as source and sink of atmospheric CO_2_ and other greenhouse gases; this dataset may be used in combination with proteomics to evaluate the microbial processes in these waters.
• This dataset can be a link to understand microbial community structure in other twilight zones of other oceans.

## 1 Objective

Southern Ocean is an environmentally isolated biome but has a large diversity and biomass of micro and macroplankton communities. The Southern Ocean is circumpolar, without any continental barriers and is linked with the Pacific, Atlantic and Indian Oceans. The strong meridional temperature and salinity gradients make it unique from the other oceans. Such stratified water masses serve as a hotspot for diverse bacterial communities including both phototrophic and heterotrophic microbes. High bacterial diversity was observed at 200 m depth in the Equatorial Indian Ocean [1]. The Southern Ocean plays a major role in flux of atmospheric carbon dioxide, organic matter, and sinking water masses [2], [3]. A strong thermocline and resultant stratification at 200 m (twilight zone) retain particulate organic matter and plankton biomass in the Indian Sector of Southern Ocean [4].

**Fig. 1:**
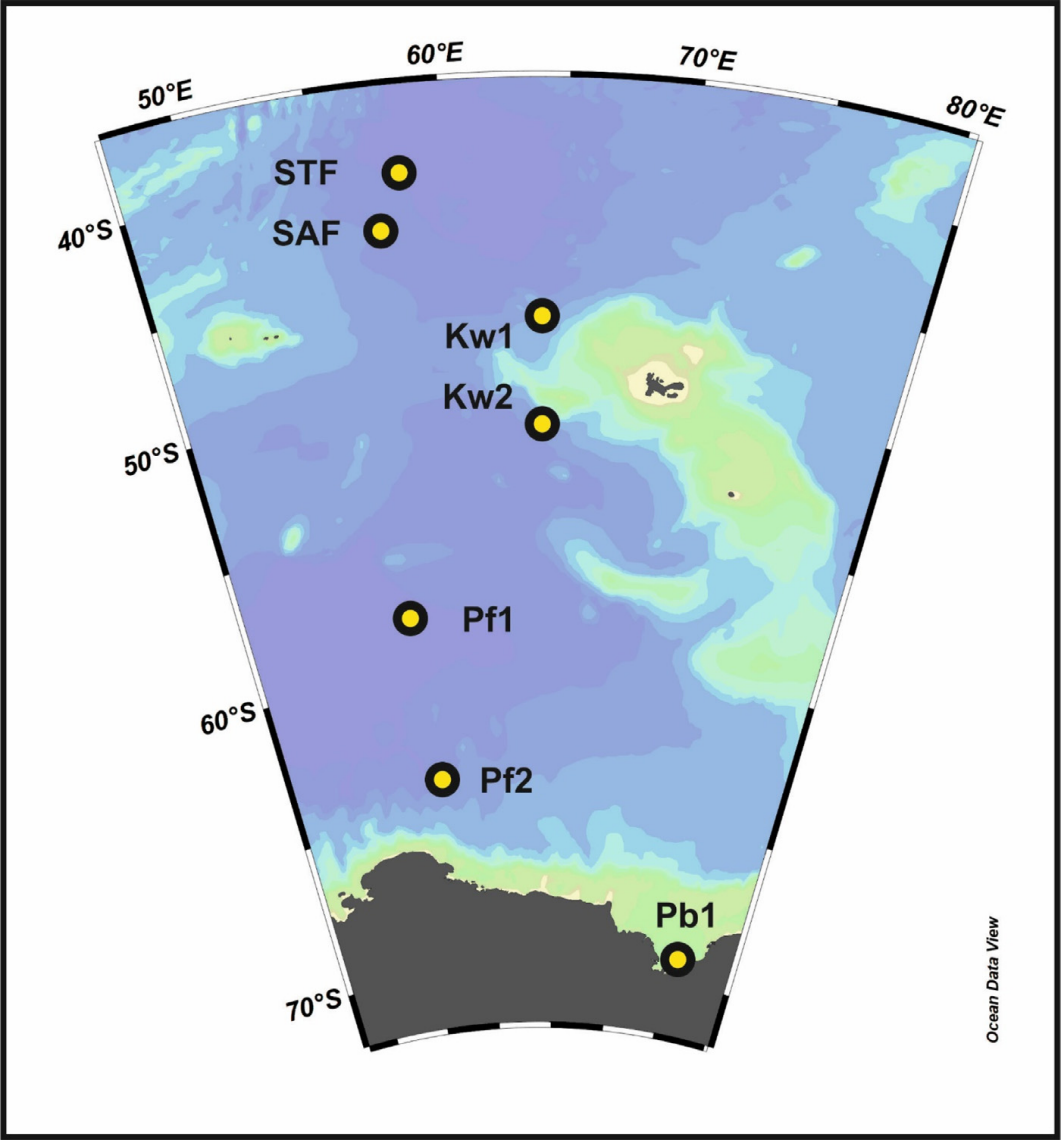
Map showing the sampling locations of the Indian Sector of Southern Ocean during ISO-9 (2016-17) (Details in Table 1).

Here we report the metagenomic dataset from samples collected from 200 m depth in the Indian sector of Southern Ocean. Amplicon bacterial diversity was performed using HiSeq method followed by bioinformatics analysis using Mothur v 1.48.0 and Silva 138.1 nr database.

## 2 Data Description

The samples collected from seven different stations in Indian Sector of the Southern Ocean (Fig. 1) were treated for DNA extraction and the hypervariable regions V3-V4 of 16s rDNA were sequenced using HiSeq sequencing technique. The sequences were then subjected to bioinformatics processing using Mothur v 1.48.0 [5]. Taxonomic classification was carried out using Silva 138.1 nr database. Further downstream processing of data was generated using MicrobiomeAnalyst [6].

**Fig. 2:**
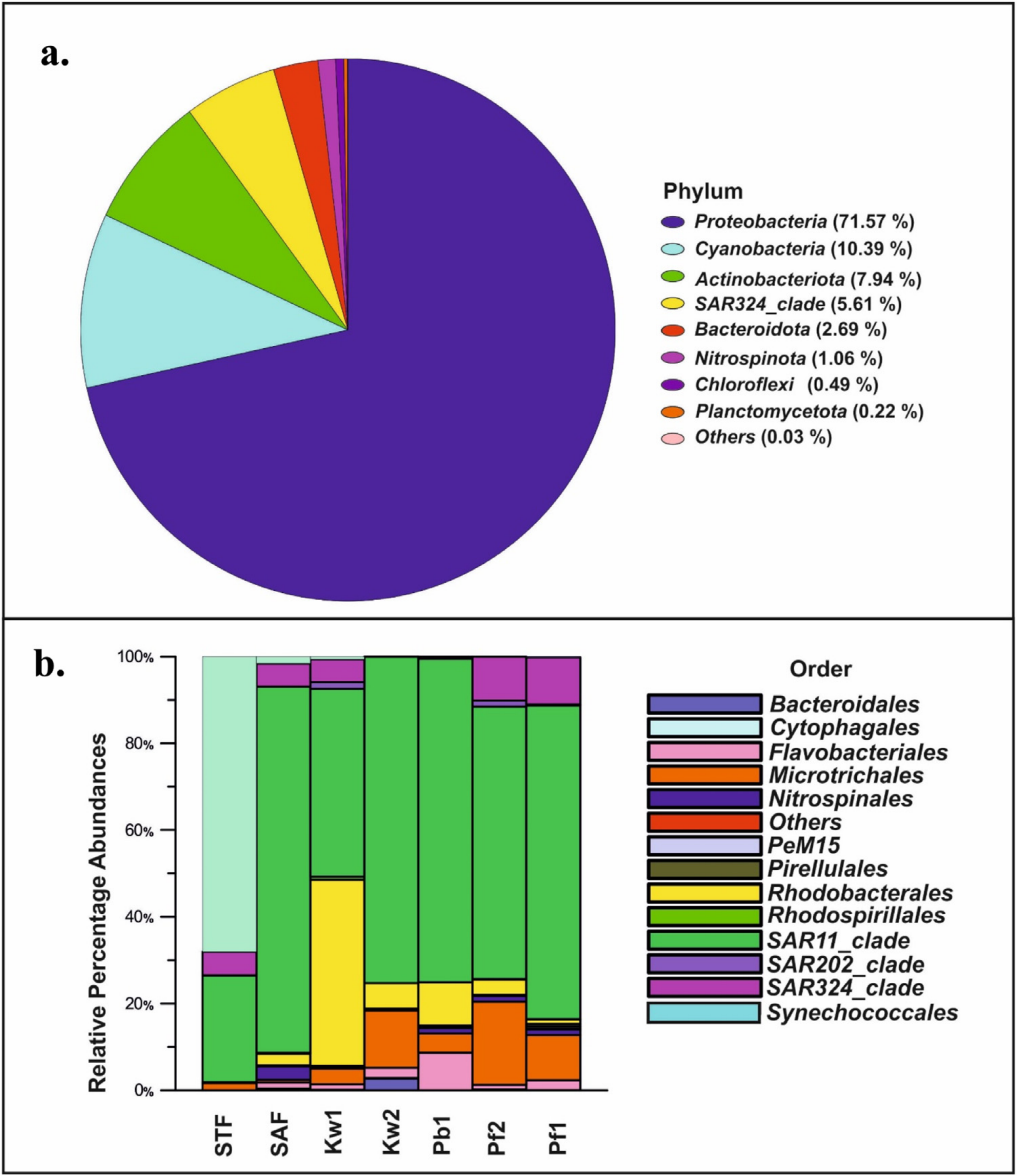
Bacterial distribution (a) representing the cumulative phylum, and (b) Relative percentage distribution at Order level from different regimes in the Indian sector of Southern Ocean during ISOE-9.

All phyla were omitted or merged with a count smaller than 10 taxa. After processing the taxonomic data, 9 different phyla were identified, with *Proteobacteria* (71.57%)*, Cyanobacteria* (10.39%)*, Actinobacteriota* (10.39%) SAR 324 clades (5.61%) *Bacteridota* (2.69%) and *Nitrospinota* (1.06%) forming the major phyla while *Chloroflexi, Planctomycetota* and *others* cumulatively constituted only 1% (Fig. 2a)*.*

At order level, *SAR 11_Clades* and *Synechococcales* were dominant in the subtropical front (STF) but *Synechococcales* were replaced with *SAR 324* clades at Subantarctic fronts (SAF), Polar fronts (PF) including Kerguelen water (KW) and Antarctic coastal waters like Prydz Bay waters (PB) (Fig. 2b).

## 3 Experimental Design, Materials and Methods

Seven different sampling locations were selected based on hydrography and nutrients variability (Table 1). Sampling was carried out onboard *SA Agulhas in austral summer,* during Indian Southern Ocean expedition 9 (ISOE-09; 2016-2017). Seawater samples were collected using a Niskin bottle (10 L sampler) (Seabird Inc., USA) attached to CTD rosette equipped with Sea bird CTD system (SBE911 plus, Sea-Bird Electronics, USA)[7]. Five litres (5 L) of water samples were filtered through 0.22-µm pore size, 47 mm diameter polycarbonate filters (Merck Millipore, USA) for bacterial diversity analysis. The filters were sealed in sterile tubes, stored at -80°C and transported to the laboratory for DNA extraction [7].

**Table 1:** Detail of sequence summary of all the samples collected from 200 m depth after sequencing and quality filtration.

Sample No	Sample ID	Latitude	Longitude	Total Read	GC contents	Phred Score
1	STF	-40.04	58.30	101082	51.90	36.91
2	SAF	-42.59	57.30	361815	49.82	37.95
3	Kw1	-46.59	64.00	909694	52.91	37.14
4	Kw2	-51.00	64.01	128583	51.20	37.82
5	Pf1	-58.03	56.49	283446	51.79	37.76
6	Pf2	-63.59	57.49	338536	51.72	37.90
7	Pb1	-69.26	75.97	169823	50.76	37.70

STF: Sub-Tropical Front; SAF: Sub-Antarctica Front; Kw: Kerguelen Waters; Pf1: Polar Front-1; Pf-2: Polar Front-2; Pb: Prydz Bay

*DNA Isolation and sequencing -* Total DNA was extracted from 0.22 μm pore-size polycarbonate membrane filter using Power Water DNA kit (MoBio; USA). The hypervariable region (V3-V4) of bacterial 16S rDNA was amplified using primer *341 F: 5’* CCTACGGGAGGCAGCAG *3’* and *806 R: 5′* GGACTACHVGGGTTCTAAT *3’* [1] with Hiseq Rapid V2 Kit for 2*250 base pair (bp) sequence. DNA sequencing was outsourced to M/s Agrigenome Laboratory Ltd. (India). The raw data of the V3-V4 sequence were deposited NCBI database. Quality scores and CG base were checked and processed for down streaming bioinformatics analysis (Table 1).

*Bioinformatics and statistics analysis-* DNA sequences were processed downstream using Mothur v-1.48.0 (Log file attached as supplementary 1). Contigs were prepared for total 2292979 reads and were trimmed, around 1739901 sequences were removed using screen.seqs command. Thereafter, 553078 sequences were selected and further 417049 sequences were selected as unique sequences. These selected unique sequences were further aligned using Silva.nr_v138.1 database. The sequential downstream processing was carried out and a total of 250842 sequences were obtained and subsampled with reference to the smallest group of 3309 sequences. Chloroplast, mitochondria, Eukaryota, Archaea and unknown samples were removed using remove.lineage command and further shared and taxonomy files were created with cutoff value of 0.03. Grapher 10, Microbiomeanalyst and R software was used for downstream processing and final data preparation.

## Ethics Statements

This article does not contain any studies with human participation or animal performed by any of the authors.

## Credit Author Statement

**Alok K. Sinha** – Sample Collection, Methodology, Data curation, Writing original draft preparation; **Bhaskar V. Parli** – Visualization, Supervision and Editing; **N. Anilkumar** – Supervision and Project Leader.

## Funding Support

This work was carried out under SOCarP project funded by Ministry of Earth Sciences (MoES), Government of India (GoI).

## Declaration of Competing Interest

The authors declare that they have no known competing financial interests or personal relationships that could have appeared to influence the work reported in this paper.

## Data Availability

Southern Ocean Deep Watesr Sample (Original data) (NCBI)

## Appendix Supplementary materials

Image, application 1
